# Voriconazole-induced liver injury: incidence patterns and risk factors in a retrospective cohort

**DOI:** 10.1128/aac.00487-25

**Published:** 2025-07-31

**Authors:** Yan Lou, Yu Wang, Jing Liu, Yu-Jing Wang, Jiaqi Wang, Shuang Ma, Zong-Ming Yang, Jing-Song Li, Yun-Qing Qiu

**Affiliations:** 1 Department of Clinical Pharmacy, Zhejiang Provincial Key Laboratory for Drug Clinical Research and Evaluation, The First Affiliated Hospital, College of Medicine, Zhejiang University 12377, Hangzhou, Zhejiang, People's Republic of China; 2 Research Center for Healthcare Data Science, Zhejiang Lab, Hangzhou, Zhejiang, People’s Republic of China; 3 Zhejiang University 12377, Hangzhou, Zhejiang, People's Republic of China; 4 Engineering Research Center of EMR and Intelligent Expert Systems, Ministry of Education, Key Laboratory for Biomedical Engineering of Ministry of Education, College of Biomedical Engineering and Instrument Science, Zhejiang University 12377, Hangzhou, Zhejiang, People's Republic of China; University of Iowa, Iowa City, Iowa, USA

**Keywords:** voriconazole, drug-induced liver injury, incidence patterns, risk factors

## Abstract

Voriconazole, a first-line therapy for invasive aspergillosis, carries hepatotoxicity risks lacking comprehensive epidemiological characterization. Current evidence is limited by heterogeneity in diagnostic criteria (e.g., varying thresholds for liver function abnormalities across studies), insufficient sample sizes, and inadequate adjustment for comorbidities, which compromises risk assessment accuracy. This study aims to systematically characterize the epidemiological profile of voriconazole-induced liver injury and identify modifiable independent risk factors. We analyzed 7,659 voriconazole-treated adults (2007–2020) from the First Affiliated Hospital of Zhejiang University to determine incidence and injury subtypes. Univariable and multivariable logistic regression identified risk factors, with model performance evaluated by ROC (receiver-operating characteristic) analysis; sensitivity analyses addressed potential confounders. In our results, voriconazole-induced liver injury occurred in 11.32% of patients, predominantly manifesting as hepatocellular (5.01%, 279/630), cholestatic (5.19%, 289/630), and mixed-pattern injuries (1.11%, 62/630). Key modifiable risk factors included intravenous administration (odds ratio [OR] = 1.88, 95% confidence interval [CI]: 1.43–2.46). Non-modifiable predictors comprised baseline elevated urea (OR = 1.47, 95% CI: 1.08–2.02), elevated triglycerides (TG) (cholestatic injury: OR = 1.47, 95% CI: 1.08–2.02), elevated C-reactive protein (CRP) (cholestatic injury: OR = 1.47, 95% CI: 1.08–2.02), pre-existing liver disease (liver cirrhosis, OR = 2.16, 95% CI: 1.58–2.96), diabetes (OR = 2.19, 95% CI: 1.70–2.80), and transplant status (hematopoietic stem cell transplantation, OR = 2.93, 95% CI: 1.85–4.65). Using this clinically substantial cohort (*n*=7,659), we proposed a risk-stratification framework incorporating administration route optimization and comorbidity-specific monitoring, supporting personalized risk-benefit analysis in antifungal therapy.

## INTRODUCTION

Drug-induced liver injury (DILI) stands as the leading cause of post-marketing drug withdrawals and acute liver failure worldwide (1, 2). Epidemiological analyses demonstrate distinct geographic patterns in DILI incidence, with disproportionate prevalence observed in Asia-Pacific populations (3).

Antimicrobial agents constituted a substantial proportion of DILI cases, with particular antimicrobial classes demonstrating elevated risks of severe hepatotoxicity (4). As the first-line therapeutic agent for invasive aspergillosis, voriconazole presents a notable safety challenge in clinical practice due to its clinically relevant liver injury, warranting systematic safety evaluation. Pharmacoepidemiologic analyses demonstrate that voriconazole-induced liver injury constitutes 12.5%–28.6% of all reported DILI events (5, 6). Notably, a study reporting 22.17% mortality (7) reflected all-cause mortality in critically ill patients with invasive fungal infections and multiorgan failure. While voriconazole hepatotoxicity is typically reversible upon discontinuation, high-risk subgroups like hematopoietic stem cell transplant (HSCT) recipients face significant therapeutic challenges: Voriconazole withdrawal due to hepatotoxicity was associated with 8.9% attributable mortality in this population (8), creating a critical risk-benefit dilemma.

Pre-existing hepatic impairment (e.g., chronic hepatitis, cirrhosis) increases susceptibility to voriconazole-induced liver injury, likely through compromised metabolic capacity leading to elevated trough concentrations (9). Bone marrow transplant recipients demonstrate a 34% incidence of voriconazole-induced liver injury (10). Concomitant medications exhibit synergistic hepatotoxic effects: proton pump inhibitors increase their incidence (11), while statin co-administration elevates the risk of alanine aminotransferase (ALT) >3 × upper limit of normal (ULN) (12). Pharmacogenetic variability, particularly CYP2C19 polymorphisms affecting voriconazole clearance, further modulates liver injury risk through pharmacokinetic alterations (13). This highlights the potential importance of pharmacokinetic individual variability in risk stratification.

Current evidence remains limited by methodological inconsistencies (14). Variability in DILI diagnostic criteria compromises incidence estimates, while inadequate adjustment for confounders (e.g., comorbidities, polypharmacy, baseline characteristics) reduces result generalizability. Moreover, independent prognostic factors identified in previous studies have not been rigorously validated through sensitivity analyses, necessitating further evaluation of the reliability and reproducibility of these results.

Based on the current research landscape, this study aims to systematically assess the incidence, clinical features, and prognostic factors of voriconazole-induced liver injury using a retrospective cohort design. By identifying and validating risk factors, this study seeks to provide robust scientific evidence to facilitate the identification of high-risk patients and the optimization of treatment regimens, ultimately reducing the incidence of voriconazole-induced liver injury and improving patient outcomes.

## MATERIALS AND METHODS

### Study population and study design

A retrospective cohort study was conducted utilizing electronic health records (EHRs) from the First Affiliated Hospital of Zhejiang University School of Medicine. This study encompassed all inpatients who underwent voriconazole therapy from September 2007 to November 2020. Exclusion criteria included patients who were treated with voriconazole for less than 1 day, as well as those under the age of 18. Additionally, patients with biochemical levels reaching the liver injury criteria mentioned below within 30 days before starting voriconazole therapy were also excluded. This exclusion was intended to ensure the accurate estimation of the incidence of new-onset voriconazole-induced liver injury, defined as the occurrence of new cases in an at-risk population during a specified time period. Excluding individuals with pre-existing liver injury helps prevent misclassification and ensures that only incident cases are captured. This approach aligns with FDA DILI guidance (FDA Guidance for Industry: Drug-Induced Liver Injury) and standard pharmacovigilance methodologies (15), and those who did not undergo liver function testing within the observation window were also excluded. The observation window spanned from the initial administration of voriconazole to 30 days post the final administration. Patients had to maintain a continuous prescription throughout this period, with no breaks longer than 14 days in each observation period. For each patient, only the first observation window was considered for analysis.

### Definition of liver injury

In this study, plasma levels of ALT, alkaline phosphatase (ALP), aspartate aminotransferase, γ-glutamyl transpeptidase (GGT), and total bilirubin (T-Bil) were utilized as key indicators for assessing liver injury. To establish a baseline for normal liver function, patients needed to show normal results in two consecutive tests for these five indicators within 30 days before starting voriconazole treatment. In line with the European Association for the Study of the Liver Clinical Practice Guidelines (16, 17), patients were categorized into the DILI group based on the following criteria observed during the study’s observation window:

1.  ALT ≥ 5 × upper limit of normal (ULN);
2.  ALT ≥ 3 × ULN and T-Bil＞2 × ULN;
3.  ALP ≥ 2 × ULN, GGT > ULN, and there was no known bone pathology driving elevated ALP levels.

In this study, the initiation date of voriconazole treatment was referred to as the index date. For patients who exhibited abnormalities in any of the five liver function indicators within 30 days prior to the index date, the last two consecutive test results before the index date were used to establish personalized baseline values. These personalized baselines replaced the standard ULN during the observation window to assess liver injury. This dynamic threshold adjustment enabled a more accurate identification of new-onset DILI by accounting for pre-existing liver dysfunction. For example, if the baseline ALT was 1.5 × ULN, a ≥5-fold increase from baseline (i.e., reaching ≥7.5 × ULN) was required to define drug-induced liver injury. Patients who did not meet these criteria were categorized into the control group, serving as the comparison cohort for identifying risk factors associated with DILI occurrence. Additionally, patients with abnormal baseline liver function—defined as having one or more of the specified indicators exceeding the upper limit of normal but not reaching hepatotoxicity thresholds—were also included in the cohort.

Drug-induced liver injury can be classified into three distinct patterns of injury: hepatocellular, cholestatic, or mixed. This classification is based on the ratio of ALT to ALP levels, expressed as multiples of the ULNs.

- A hepatocellular pattern is defined as a ratio ≥5.
- A cholestatic pattern is defined as a ratio ≤2.
- A mixed pattern is identified when the ratio falls between 2 and 5.

### Clinical data collection

To investigate the risk factors associated with voriconazole-induced liver injury, we meticulously extracted baseline variables from the EHRs of the First Affiliated Hospital of Zhejiang University School of Medicine. These variables encompassed multiple dimensions to ensure a comprehensive analysis of the factors influencing the outcomes of voriconazole therapy. The study included demographic factors such as age and gender, as well as detailed information about the patient’s visit, including the department of the current visit. Specifics of voriconazole treatment, including average dosage per day, duration of therapy, and administration method, were also thoroughly examined. Additionally, an extensive review of pre-medication laboratory tests was conducted, covering blood lipid-related indicators, kidney function indicators, and other laboratory test indicators. The presence of comorbidities and the use of types of combination medications were meticulously recorded and analyzed.

Age, gender, visit department, and allergy history information were obtained from hospitalization summaries. Administration details of voriconazole were extracted differently for patients in the DILI group and the control group. For the DILI group, administration information from the index date to the date of liver injury occurrence was considered. For the control group, administration information throughout the entire observation window was extracted. Pre-medication laboratory test results included the most recent tests conducted within 30 days before the index date. Information on comorbidities was derived from structured diagnosis data, medical notes, and specific diagnostic laboratory results.

Besides, combination medications considered in this study focused on other hepatotoxic drugs, which were classified systematically into several major groups: nonsteroidal anti-inflammatory drugs (NSAIDs), anti-infective drugs, anti-tumor drugs, drugs for the central nervous system, cardiovascular drugs, drugs for metabolic diseases, hormonal drugs, biopharmaceuticals, and traditional Chinese medicine-natural medicine-health products-dietary supplements (TCM-NM-HP-DS). This classification was informed by current guidelines and literature, acknowledging that many drugs may exhibit hepatotoxicity at varying incidences. The specific drugs included in this study are shown in Table S1. If patients received prescriptions of the aforementioned medications within 21 days before the index date, they were categorized as receiving combination medications. All baseline variables were converted into categorical data for analysis, with the specifics of data collection detailed in Table S1.

### Statistics analysis

The incidence rate, as the number of DILI cases divided by the total subjects at risk, and the cumulative incidence of voriconazole-induced liver injury were calculated. To investigate the risk factors, we employed univariate logistic regression to calculate crude and age- and gender-adjusted odds ratio (OR) for the association of individual demographic and clinical characteristics with the risk of voriconazole-induced liver injury. The primary analysis involved a multivariate backward logistic regression approach, incorporating age, gender, and variables demonstrating significance in the age- and gender-adjusted univariate analysis. Collinearity was assessed prior to variable inclusion using the variance inflation factor (VIF), with a threshold of VIF <5 indicating acceptable multicollinearity. Backward elimination was subsequently applied for final variable selection at a significance level of 0.05. We used all independent factors to construct an additional logistic regression model and calculated the area under the receiver operating characteristic curve (AUC) to assess model performance.

Two sets of sensitive analyses were undertaken. Firstly, considering the risk of liver injury in individuals with pre-existing liver conditions, we excluded patients exhibiting abnormal baseline liver function indicators. This exclusion allowed for a more focused analysis of the risk factors contributing to liver injury incidence in patients with normal liver function. Secondly, we investigated the risk factors associated with DILI in various contexts of the voriconazole administration, including the route of voriconazole administration, therapy duration, average daily dosage, and cumulative dosage. Backward logistic regression models were constructed respectively to elucidate the independent risk factors within these four specified contexts, and receiver-operating characteristic (ROC) curves were plotted.

Statistical analyses were performed using SAS version 9.4 (SAS Inc.). For all statistical tests, we employed a two-sided approach, and differences with a *P*-value <0.05 were considered statistically significant.

## RESULTS

### Incidence and subtype distribution of voriconazole-induced liver injury

**Fig 1:**
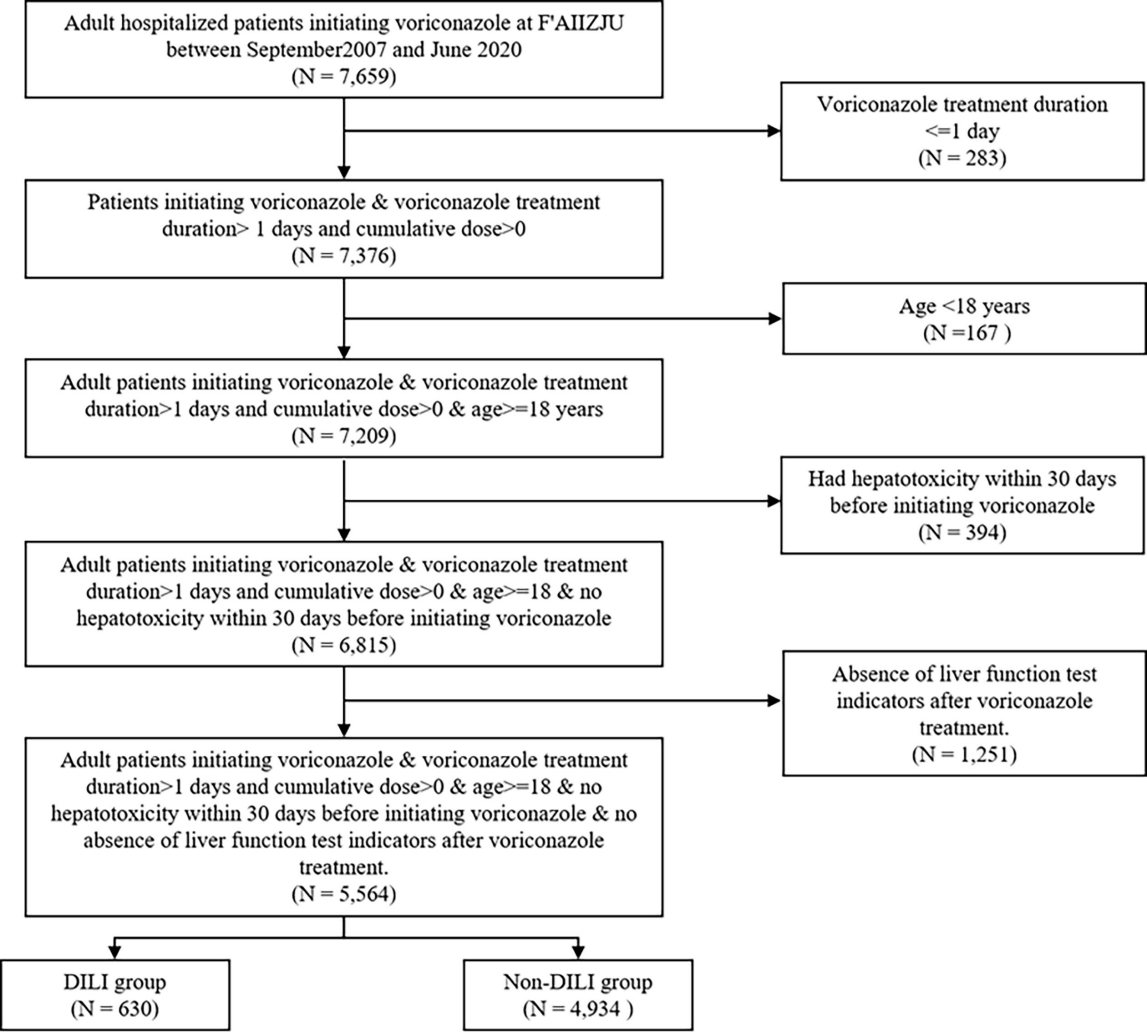
The flowchart of the study population.

The study cohort comprised 7,659 voriconazole-treated patients (2007–2020), with 5,564 meeting inclusion criteria after rigorous screening (Fig. 1). The confirmed liver injury cases (*n* = 630, 11.32%) demonstrated phenotype-specific distributions: hepatocellular (5.01%, 279/630), cholestatic (5.19%, 289/630), and mixed patterns (1.11%, 62/630). Regarding recovery from liver test abnormalities, we defined biochemical improvement as >50% reduction in ALT within 30–60 days or ALP within 180 days post-discontinuation. Among 556 evaluable DILI patients, 376 (67.6%) achieved complete/partial recovery—aligning with established antifungal-DILI resolution rates (8). The patients younger than 60 years accounted for a large proportion (56.7%), with 13.8% suffering from DILI. Among the DILI cases, the proportions of patients with DILI were similar between males and females.

Stratified analysis demonstrated treatment duration-dependent variations in DILI incidence (Table 1): higher rates occurred in patients receiving short-term therapy (<1 week: 15.64%; 1–2 weeks: 13.55%) versus prolonged durations (2–4 weeks: 9.99%; >4 weeks: 6.45%). While an inverse association between treatment duration and DILI incidence was observed, we acknowledge potential confounding from early discontinuation in hepatotoxicity cases. Table S2 provides a more descriptive analysis of voriconazole treatment duration and cumulative voriconazole treatment duration concerning DILI incidence after the baseline time point. The incidence of DILI also varied depending on the administration method: intravenous route was associated with the highest DILI incidence (18.79%), contrasting with oral (7.55%) and combined routes (8.95%). Dose-response analysis revealed complex patterns: low daily doses (≤200 mg) correlated with elevated hepatocellular injury (11.23%, 54/75), whereas high doses (>400 mg) increased cholestatic manifestations (7.20%, 19/49). Cumulative dose stratification demonstrated maximal DILI risk at low exposure levels (<1.5 g: 21.98%), decreasing with higher cumulative doses (≥3 g: 9.91%).

**TABLE 1:** Incidence of DILI and its subtypes in hospitalized patients initiating voriconazole

Variable^*a*^	Number of participants (n)	DILI (n,%)	Hepatocellular	Cholestatic	Mixed
Total	5,564	630 (11.32)	279 (5.01)	289 (5.19)	62 (1.11)
Age (years)					
<40	1,154	185 (16.03)	97 (8.41)	69 (5.98)	19 (1.65)
40~	2,002	251 (12.54)	108 (5.39)	120 (5.99)	23 (1.15)
≥60	2,408	194 (8.06)	74 (3.07)	100 (4.15)	20 (0.83)
Gender					
Male	3,673	424 (11.54)	191 (5.20)	189 (5.15)	44 (1.20)
Female	1,891	206 (10.89)	88 (4.65)	100 (5.29)	18 (0.95)
Department					
Hematology dept	2,392	227 (9.49)	105 (4.39)	99 (4.14)	23 (0.96)
Infection dept	1,033	85 (8.23)	28 (2.71)	44 (4.26)	13 (1.26)
Pneumology dept	642	39 (6.07)	15 (2.34)	22 (3.43)	2 (0.31)
Intensive care unit	474	97 (20.46)	40 (8.44)	47 (9.92)	10 (2.11)
Hepatopancreatobiliary surgery dept	202	113 (55.94)	73 (36.14)	35 (17.33)	5 (2.48)
Other depts	821	69 (8.40)	18 (2.19)	42 (5.12)	9 (1.10)
Administration route					
Oral	2,080	157 (7.55)	68 (3.27)	73 (3.51)	16 (0.77)
Intravenous	1,671	314 (18.79)	155 (9.28)	124 (7.42)	35 (2.09)
Oral + intravenous	1,766	158 (8.95)	55 (3.11)	92 (5.21)	11 (0.62)
VCZ dose per day					
≤200 mg	481	75 (15.59)	54 (11.23)	18 (3.74)	3 (0.62)
200~ mg	4,819	506 (10.50)	199 (4.13)	252 (5.23)	55 (1.14)
>400 mg	264	49 (18.56)	26 (9.85)	19 (7.20)	4 (1.52)
VCZ cumulative time					
≤1 week	1,407	220 (15.64)	139 (9.88)	65 (4.62)	16 (1.14)
1–2 weeks	1,269	172 (13.55)	62 (4.89)	79 (6.23)	31 (2.44)
2–4 weeks	1,461	146 (9.99)	47 (3.22)	93 (6.37)	6 (0.41)
>4 weeks	1,427	92 (6.45)	31 (2.17)	52 (3.64)	9 (0.63)
VCZ cumulative dose					
<1.5 g	687	151 (21.98)	105 (15.28)	36 (5.24)	10 (1.46)
1.5–3.0 g	933	88 (9.43)	40 (4.29)	40 (4.29)	8 (0.86)
≥3 g	3,944	391 (9.91)	134 (3.40)	213 (5.40)	44 (1.12)
Prior drug allergies					
No	5,125	586 (11.43)	253 (4.94)	276 (5.39)	57 (1.11)
Yes	439	44 (10.02)	26 (5.92)	13 (2.96)	5 (1.14)
Albumin					
<LLN	2,093	204 (9.75)	74 (3.54)	109 (5.21)	21 (1.00)
Normal	2,536	270 (10.65)	137 (5.40)	106 (4.18)	27 (1.06)
>ULN	3	1 (33.33)	0 (0.00)	1 (33.33)	0 (0.00)
Missing	932	155 (16.63)	68 (7.30)	73 (7.83)	14 (1.50)
Globulin					
<LLN	986	132 (13.39)	58 (5.88)	55 (5.58)	19 (1.93)
Normal	4,104	454 (11.06)	201 (4.90)	212 (5.17)	41 (1.00)
>ULN	474	44 (9.28)	20 (4.22)	22 (4.64)	2 (0.42)
HDL					
<LLN	1,867	231 (12.37)	101 (5.41)	109 (5.84)	21 (1.12)
Normal	2,435	209 (8.58)	99 (4.07)	87 (3.57)	23 (0.94)
>ULN	117	5 (4.27)	2 (1.71)	1 (0.85)	2 (1.71)
Missing	1,145	185 (16.16)	77 (6.72)	92 (8.03)	16 (1.40)
LDL					
<LLN	927	132 (14.24)	52 (5.61)	69 (7.44)	11 (1.19)
Normal	3,027	272 (8.99)	128 (4.23)	110 (3.63)	34 (1.12)
>ULN	457	41 (8.97)	22 (4.81)	18 (3.94)	1 (0.22)
Missing	1,153	185 (16.05)	77 (6.68)	92 (7.98)	16 (1.39)
TC					
<LLN	1,380	169 (12.25)	74 (5.36)	79 (5.72)	16 (1.16)
Normal	2,737	248 (9.06)	117 (4.27)	102 (3.73)	29 (1.06)
>ULN	304	28 (9.21)	11 (3.62)	16 (5.26)	1 (0.33)
Missing	1,143	185 (16.19)	77 (6.74)	92 (8.05)	16 (1.40)
URICACID					
<LLN	1,830	229 (12.51)	96 (5.25)	111 (6.07)	22 (1.20)
Normal	2,541	221 (8.70)	101 (3.97)	98 (3.86)	22 (0.87)
>ULN	602	74 (12.29)	31 (5.15)	37 (6.15)	6 (1.00)
Missing	591	106 (17.94)	51 (8.63)	43 (7.28)	12 (2.03)
Urea					
<LLN	469	50 (10.66)	21 (4.48)	25 (5.33)	4 (0.85)
Normal	3,419	327 (9.56)	160 (4.68)	138 (4.04)	29 (0.85)
>ULN	1,088	147 (13.51)	47 (4.32)	83 (7.63)	17 (1.56)
Missing	588	106 (18.03)	51 (8.67)	43 (7.31)	12 (2.04)
Creatinine					
<LLN	1,389	191 (13.75)	84 (6.05)	90 (6.48)	17 (1.22)
Normal	2,839	243 (8.56)	117 (4.12)	100 (3.52)	26 (0.92)
>ULN	748	90 (12.03)	27 (3.61)	56 (7.49)	7 (0.94)
Missing	588	106 (18.03)	51 (8.67)	43 (7.31)	12 (2.04)
CRP					
Normal	1,862	187 (10.04)	108 (5.80)	60 (3.22)	19 (1.02)
>ULN	3,702	443 (11.97)	171 (4.62)	229 (6.19)	43 (1.16)
Liver disease					
No	4,108	390 (9.49)	156 (3.80)	189 (4.60)	45 (1.10)
Yes	1,456	240 (16.48)	123 (8.45)	100 (6.87)	17 (1.17)
Hepatitis					
No	4,621	469 (10.15)	186 (4.03)	233 (5.04)	50 (1.08)
Yes	943	161 (17.07)	93 (9.86)	56 (5.94)	12 (1.27)
Viral hepatitis					
No	5,003	524 (10.47)	216 (4.32)	255 (5.10)	53 (1.06)
Yes	561	106 (18.89)	63 (11.23)	34 (6.06)	9 (1.60)
Fatty liver					
No	5,170	574 (11.10)	256 (4.95)	260 (5.03)	58 (1.12)
Yes	394	56 (14.21)	23 (5.84)	29 (7.36)	4 (1.02)
Liver cirrhosis					
No	5,124	509 (9.93)	206 (4.02)	248 (4.84)	55 (1.07)
Yes	440	121 (27.50)	73 (16.59)	41 (9.32)	7 (1.59)
Liver cancer					
No	5,513	607 (11.01)	260 (4.72)	286 (5.19)	61 (1.11)
Yes	51	23 (45.10)	19 (37.25)	3 (5.88)	1 (1.96)
Diabetes mellitus					
No	3,973	362 (9.11)	157 (3.95)	165 (4.15)	40 (1.01)
Yes	1,188	206 (17.34)	98 (8.25)	94 (7.91)	14 (1.18)
Unknown	403	62 (15.38)	24 (5.96)	30 (7.44)	8 (1.99)
Hyperlipidemia					
No	5,336	609 (11.41)	269 (5.04)	281 (5.27)	59 (1.11)
Yes	228	21 (9.21)	10 (4.39)	8 (3.51)	3 (1.32)
Hypertension					
No	4,254	511 (12.01)	239 (5.62)	217 (5.10)	55 (1.29)
Yes	1,310	119 (9.08)	40 (3.05)	72 (5.50)	7 (0.53)
Hematological diseases					
No	2,965	372 (12.55)	159 (5.36)	177 (5.97)	36 (1.21)
Yes	2,599	258 (9.93)	120 (4.62)	112 (4.31)	26 (1.00)
Leukemia					
No	3,990	442 (11.08)	185 (4.64)	215 (5.39)	42 (1.05)
Yes	1,574	188 (11.94)	94 (5.97)	74 (4.70)	20 (1.27)
Lymphoma					
No	4,674	578 (12.37)	262 (5.61)	260 (5.56)	56 (1.20)
Yes	890	52 (5.84)	17 (1.91)	29 (3.26)	6 (0.67)
Myeloma					
No	5,144	591 (11.49)	263 (5.11)	267 (5.19)	61 (1.19)
Yes	420	39 (9.29)	16 (3.81)	22 (5.24)	1 (0.24)
Myelodysplastic syndrome					
No	5,338	599 (11.22)	265 (4.96)	276 (5.17)	58 (1.09)
Yes	226	31 (13.72)	14 (6.19)	13 (5.75)	4 (1.77)
Transplant state					
No	5,291	570 (10.77)	259 (4.90)	258 (4.88)	53 (1.00)
Yes	273	60 (21.98)	20 (7.33)	31 (11.36)	9 (3.30)
Hematopoietic stem cell transplantation					
No	5,435	599 (11.02)	264 (4.86)	277 (5.10)	58 (1.07)
Yes	129	31 (24.03)	15 (11.63)	12 (9.30)	4 (3.10)
Organ transplantation					
No	5,418	600 (11.07)	274 (5.06)	269 (4.96)	57 (1.05)
Yes	146	30 (20.55)	5 (3.42)	20 (13.70)	5 (3.42)
Kidney transplantation					
No	5,470	617 (11.28)	276 (5.05)	282 (5.16)	59 (1.08)
Yes	94	13 (13.83)	3 (3.19)	7 (7.45)	3 (3.19)
Liver transplantation					
No	5,520	614 (11.12)	278 (5.04)	276 (5.00)	60 (1.09)
Yes	44	16 (36.36)	1 (2.27)	13 (29.55)	2 (4.55)
Lung transplantation					
No	5,558	629 (11.32)	279 (5.02)	289 (5.20)	61 (1.10)
Yes	6	1 (16.67)	0 (0.00)	0 (0.00)	1 (16.67)
Bone transplantation					
No	5,563	630 (11.32)	279 (5.02)	289 (5.20)	62 (1.11)
Yes	1	0 (0.00)	0 (0.00)	0 (0.00)	0 (0.00)
Corneal transplantation					
No	5,562	630 (11.33)	279 (5.02)	289 (5.20)	62 (1.11)
Yes	2	0 (0.00)	0 (0.00)	0 (0.00)	0 (0.00)
Heart transplantation					
No	5,563	630 (11.32)	279 (5.02)	289 (5.20)	62 (1.11)
Yes	1	0 (0.00)	0 (0.00)	0 (0.00)	0 (0.00)
Gut transplantation					
No	5,561	628 (11.29)	278 (5.00)	288 (5.18)	62 (1.11)
Yes	3	2 (66.67)	1 (33.33)	1 (33.33)	0 (0.00)
NSAIDs					
No	5,202	593 (11.40)	273 (5.25)	260 (5.00)	60 (1.15)
Yes	362	37 (10.22)	6 (1.66)	29 (8.01)	2 (0.55)
Anti-infective drugs					
No	2,343	311 (13.27)	145 (6.19)	137 (5.85)	29 (1.24)
Yes	3,221	319 (9.90)	134 (4.16)	152 (4.72)	33 (1.02)
Anti-tumor drugs					
No	4,497	496 (11.03)	195 (4.34)	249 (5.54)	52 (1.16)
Yes	1,067	134 (12.56)	84 (7.87)	40 (3.75)	10 (0.94)
Drugs for central nervous system					
No	4,873	539 (11.06)	245 (5.03)	243 (4.99)	51 (1.05)
Yes	691	91 (13.17)	34 (4.92)	46 (6.66)	11 (1.59)
Cardiovascular drugs					
No	4,717	533 (11.30)	250 (5.30)	231 (4.90)	52 (1.10)
Yes	847	97 (11.45)	29 (3.42)	58 (6.85)	10 (1.18)
Drugs for metabolic diseases					
No	4,492	465 (10.35)	223 (4.96)	199 (4.43)	43 (0.96)
Yes	1,072	165 (15.39)	56 (5.22)	90 (8.40)	19 (1.77)
Hormonal drugs					
No	5,488	625 (11.39)	276 (5.03)	287 (5.23)	62 (1.13)
Yes	76	5 (6.58)	3 (3.95)	2 (2.63)	0 (0.00)
Biopharmaceutical					
No	5,555	628 (11.31)	279 (5.02)	287 (5.17)	62 (1.12)
Yes	9	2 (22.22)	0 (0.00)	2 (22.22)	0 (0.00)
TCM-NM-HP-DS					
No	5,506	625 (11.35)	277 (5.03)	288 (5.23)	60 (1.09)
Yes	58	5 (8.62)	2 (3.45)	1 (1.72)	2 (3.45)

^*a*^ VCZ, voriconazole; LLN, lower limit of normal; HDL, high-density lipoprotein; LDL, low-density lipoprotein; TC, total cholesterol; TG, triglycerides; URICACID, uric acid; CRP, C-reactive protein.

Comorbidity profiling identified substantially elevated DILI incidence in patients with liver disease (16.48%). In addition, 273 patients (4.9%) underwent some form of transplantation surgery (21.98% suffered from DILI), including both solid-organ transplants and HSCTs shown in Table 1, with liver and kidney transplants being relatively more common among this group. It is noteworthy that the overall number of patients who underwent HSCT was comparable to the number of patients who received solid-organ transplants. The incidence of DILI in patients with diabetes mellitus is relatively high (17.34%). Despite our efforts, specific reasons for voriconazole therapy and the types of fungal diseases treated could not be reliably ascertained from our EHR system.

### Multidimensional risk factor profiling

**Fig 2:**
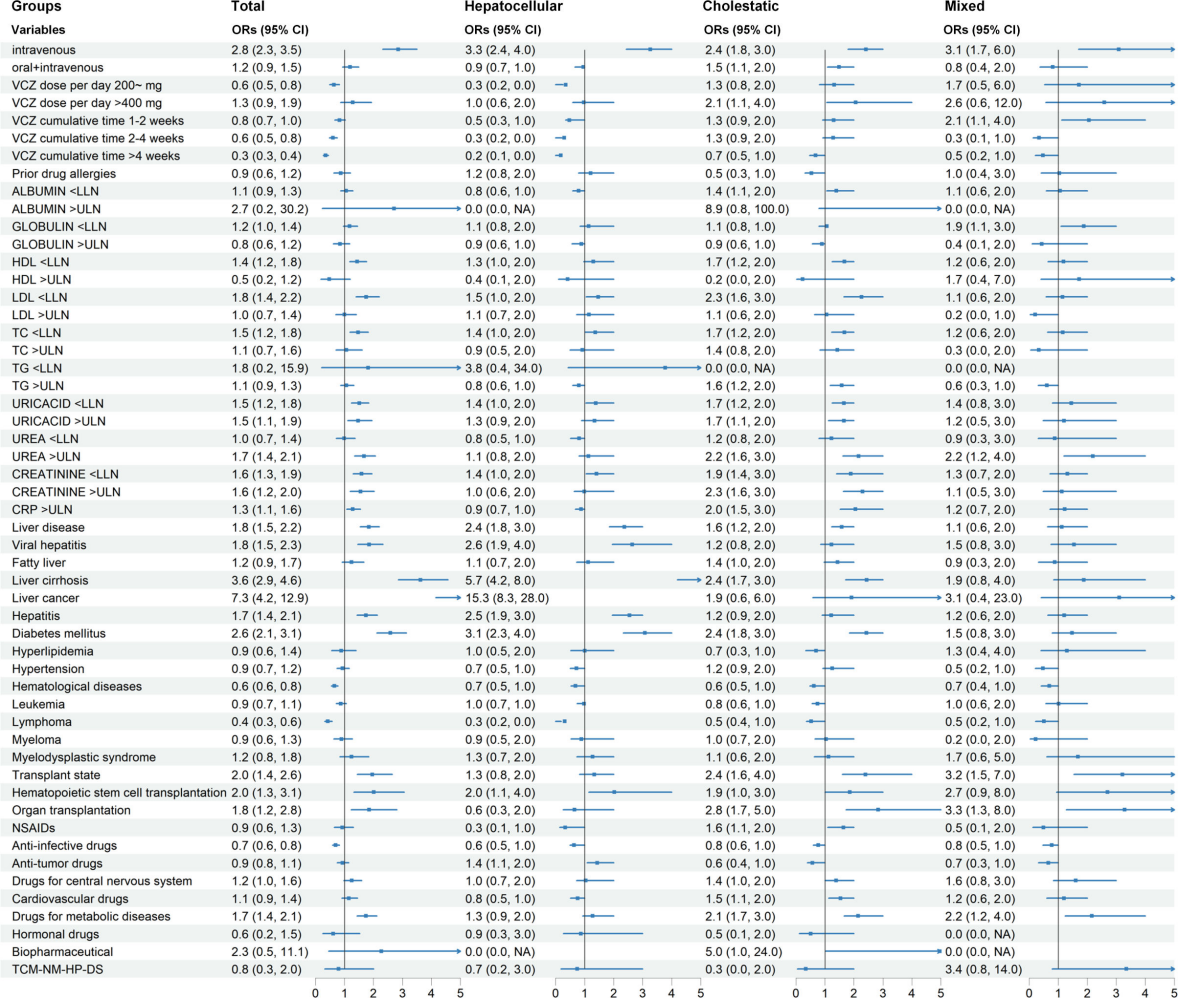
Age- and gender-adjusted univariable odds ratios for clinically significant risk factors (complete dataset in Fig. S1). Abbreviations: VCZ, voriconazole; HDL, high-density lipoprotein; LDL, low-density lipoprotein; TC, total cholesterol; TG, triglycerides; URICACID, uric acid; CRP, C-reactive protein. Reference levels: administration route: oral; VCZ dose per day: ≤200 mg; VCZ cumulative time: ≤1 week; prior drug allergies, liver disease, viral hepatitis, fatty liver, liver cirrhosis, liver cancer, hepatitis, diabetes mellitus, hyperlipidemia, hypertension, hematological diseases, leukemia, lymphoma, myeloma, myelodysplastic syndrome, transplant state, hematopoietic stem cell transplantation, organ transplantation, NSAIDs, anti-infective drugs, anti-tumor drugs, drugs for central nervous system, cardiovascular drugs, drugs for metabolic diseases, hormonal drugs, biopharmaceutical and TCM-NM-HP-DS: no; albumin, globulin, HDL, LDL, TC, TG, URICACID, urea, creatinine, and CRP: normal.

**Fig 3:**
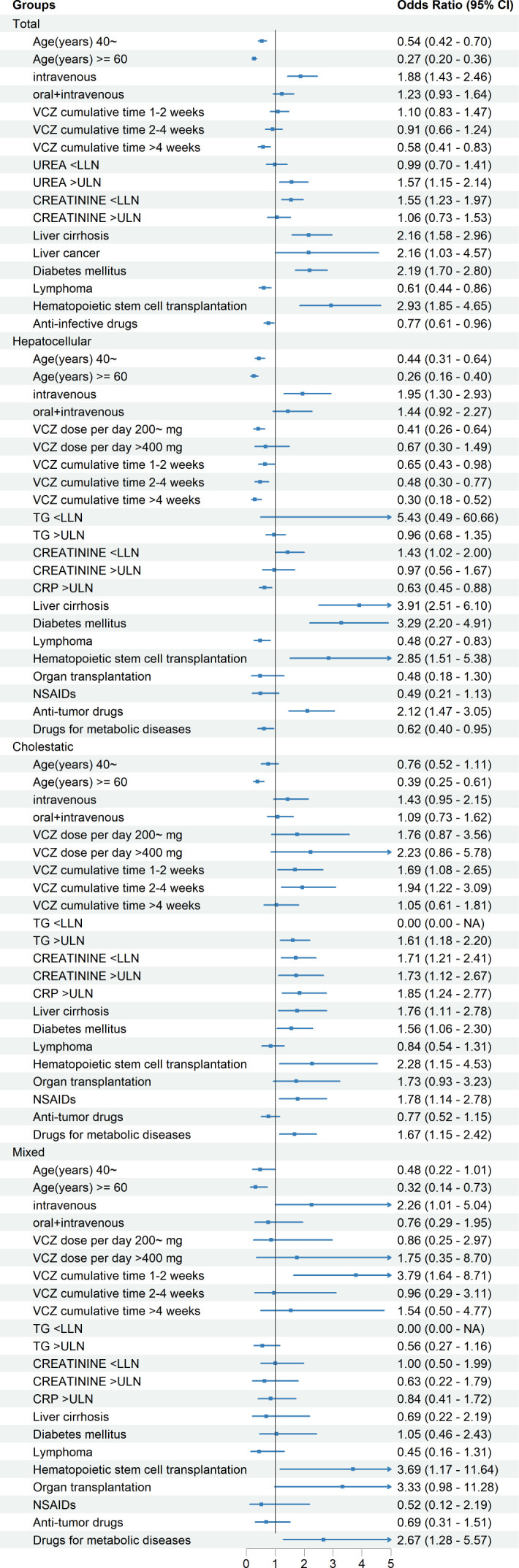
Multivariable ORs for risk factors associated with DILI and its subtypes. Abbreviations: VCZ: voriconazole; TG: triglycerides; CRP: C-reactive protein; reference levels: age (years): <40; gender: male; administration route: oral; VCZ dose per day: ≤200 mg; VCZ cumulative time: ≤1 week; liver cirrhosis, liver cancer, diabetes mellitus, lymphoma, hematopoietic stem cell transplantation, organ transplantation, anti-infective drugs, NSAIDs, anti-tumor drugs, and drugs for metabolic diseases: no; urea, TG, creatinine, and CRP: normal.

We conducted parallel univariate analyses to evaluate risk factors associated with voriconazole-induced liver injury. Specifically, we performed an age- and gender-adjusted analysis accounting for these fundamental covariates (Fig. 2). This dual approach follows current methodological recommendations (18) for comprehensive risk factor assessment. Additionally, Fig. S1 presents complete univariate results including age and gender analyses. While these demographic factors showed no significant correlation with the risk of DILI in univariate testing, we retained them in multivariate modeling due to their established biological plausibility in drug metabolism. We then conducted a multivariate logistic regression analysis of the candidate variables described in Table 1 to identify the main causes of voriconazole-induced liver injury (Fig. 2 and 3).

Intravenous administration exhibited an increased risk of total DILI (OR = 1.88, 95% confidence interval [CI]: 1.43–2.46), and also showed independent associations across hepatic injury subtypes: hepatocellular (OR = 1.95, 95% CI: 1.30–2.93) and mixed-pattern (OR = 2.26, 95% CI: 1.01–5.04). Temporal analysis revealed time-dependent cholestatic injury susceptibility, with maximal risk during early treatment phases (1–2 weeks: OR = 1.69, 95% CI: 1.08–2.65; 2–4 weeks: OR = 1.94, 95% CI: 1.22–3.09).

Comorbidity profiling identified baseline hepatic dysfunction (liver cirrhosis, OR = 2.16, 95% CI: 1.58–2.96), diabetes mellitus (OR = 2.19, 95% CI: 1.70–2.80), and transplantation status (hematopoietic stem cell transplantation, OR = 2.93, 95% CI: 1.85–4.65) as significant risk factors, and showed similar results in different subtypes of liver injury (Fig. 3).

Combination with anti-tumor drugs preferentially exacerbated hepatocellular injury (OR = 2.12, 95% CI: 1.47–3.05). Combination with NSAIDs (OR = 1.78, 95% CI: 1.14–2.78) or drugs for metabolic diseases (OR = 1.67, 95% CI: 1.15–2.42) exhibited cholestatic predisposition. Biochemical predictors included urea level elevation (overall OR = 1.57, 95% CI: 1.15–2.14) and hypocreatininemia (overall OR = 1.55, 95% CI: 1.23–1.97), the latter also correlating with cholestatic manifestations (OR = 1.73, 95% CI: 1.12–2.67). Although low creatinine may correlate with reduced muscle mass or malnutrition, albumin levels of patients showed no significant association with DILI in univariate analysis. Meanwhile, weight data were rarely documented (<5% completeness), precluding formal nutritional status assessment. Thus, the hypocreatininemia-DILI association may reflect altered renal clearance affecting voriconazole elimination rather than nutritional status, though residual confounding cannot be excluded. Both elevated C-reactive protein (CRP) (OR = 1.85, 95% CI: 1.24–2.77) and triglycerides (TG) (OR = 1.61, 95% CI: 1.18–2.20) levels were correlating with increased risks of cholestatic manifestations. This risk stratification framework delineates complex interactions between administration parameters, metabolic comorbidities, and biochemical profiles in voriconazole-induced liver injury.

The multivariable model demonstrated discriminative capacity for overall liver injury (AUC = 0.73), with subtype-specific performance: hepatocellular (AUC = 0.79), cholestatic (AUC = 0.74), and mixed (AUC = 0.79) (Fig. 5a). These results support clinical utility in subtype-stratified risk prediction.

### Sensitivity analysis of risk factor robustness

**Fig 4:**
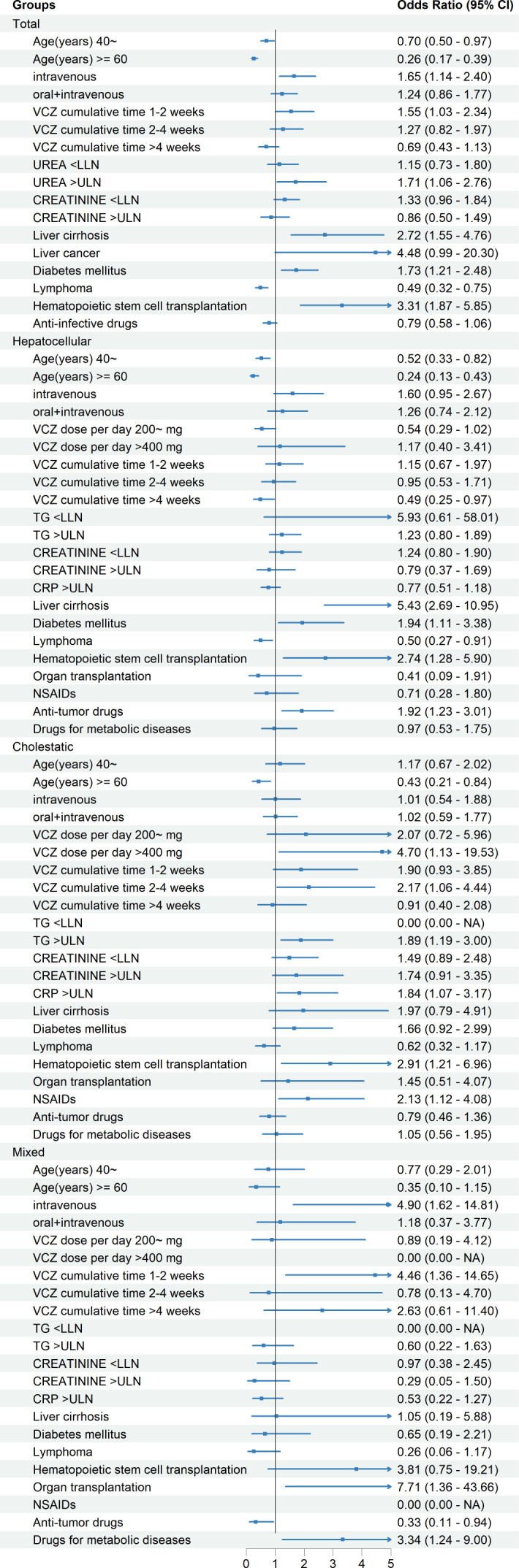
Multivariable ORs for DILI types in the hepatotoxicity group within normal baseline liver functions. Abbreviations: VCZ: voriconazole; reference levels: age (years): <40; administration route: oral; VCZ dose per day: ≤200 mg; VCZ cumulative time: ≤1 week; liver cirrhosis, liver cancer, diabetes mellitus, lymphoma, hematopoietic stem cell transplantation, organ transplantation, anti-infective drugs, NSAIDs, anti-tumor drugs, and drugs for metabolic diseases: no; urea, TG, creatinine, and CRP: normal.

Multivariable analysis of 3,784 patients with baseline normal hepatic function identified intravenous administration (adjusted OR = 1.65, 95% CI: 1.14–2.40), urea level elevation (OR = 1.71, 95% CI: 1.06–2.76), pre-existing liver disease (liver cirrhosis, OR = 2.72, 95% CI: 1.55–4.76), diabetes mellitus (OR = 1.73, 95% CI: 1.21–2.48), and transplantation history (hematopoietic stem cell transplantation, OR = 3.31, 95% CI: 1.87–5.85) as persistent risk factors for voriconazole-induced liver injury. Notably, baseline liver disease (especially liver cirrhosis) (OR = 5.43, 95% CI: 2.69–10.95) and diabetes mellitus (OR = 1.94, 95% CI: 1.11–3.38) demonstrated amplified hepatocellular injury susceptibility, suggesting hepatic functional reserve modulates toxicity mechanisms. Similarly, elevated CRP (OR = 1.84, 95% CI: 1.07–3.17) and TG levels (OR = 1.89, 95% CI: 1.19–3.00) demonstrated a preferential association with cholestatic injury risk, suggesting potential synergistic involvement of systemic inflammation and lipid dysregulation in the pathogenesis of DILI (Fig. 4).

**Fig 5:**
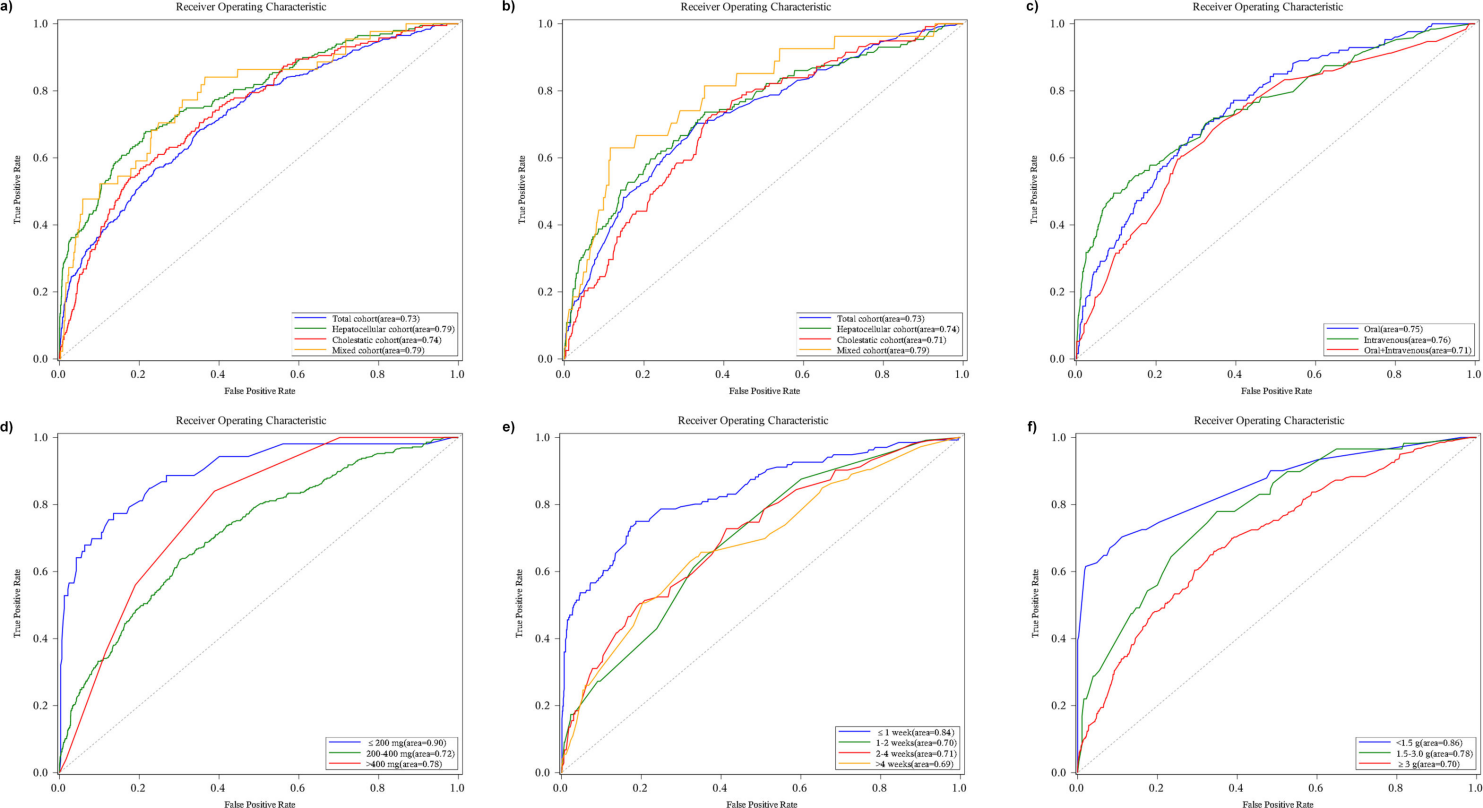
The receiver operating characteristic (ROC) curve assesses the model performance across various parameters. (a) For different types of DILI, encompassing total liver injury, hepatocellular, cholestatic, and mixed patterns; (b) among patients with normal baseline liver functions; (c) concerning types of administration routes, including oral, intravenous, and a combination of oral and intravenous; (d) considering levels of voriconazole dose per day, categorized as ≤200 mg, 200 to 400 mg, and >400 mg; (e) based on the duration of voriconazole cumulative time, categorized as ≤1 week, 1 to 2 weeks, 2 to 4 weeks, and >4 weeks; and (f) about levels of voriconazole cumulative dose, categorized as ≤1.5 g, 1.5 to 3 g, and ≥3 g.

Predictive model performance exhibited population-specific variability, with reduced discriminative capacity for overall injury (AUC = 0.73) contrasting with enhanced mixed-pattern injury prediction (AUC = 0.79; Fig. 5b), reflecting subtype-dependent pathophysiological heterogeneity in this subpopulation.

### Administration parameter stratification analysis

Stratified ROC analyses across dosing parameters revealed distinct predictive performance patterns (Fig. 5c through f). Intravenous administration demonstrated enhanced discriminative capacity (AUC = 0.76) compared to oral (AUC = 0.75) and combined routes (AUC = 0.71), indicating that intravenous administration enhances hepatic injury discrimination accuracy, potentially through circumventing first-pass metabolism and avoiding gastrointestinal metabolic interference, thereby more directly reflecting intrinsic drug hepatotoxicity. Further analysis of dosing regimens identified ≤200 mg/day as the optimal therapeutic window (AUC = 0.90), with progressive model performance decline at higher doses (200 mg–400 mg: AUC = 0.72), while doses >400 mg improved again, AUC = 0.78, consistent with metabolic saturation effects enhancing model recognition at lower doses. Temporal analysis demonstrated superior early-phase detection capability (cumulative dose ≤1 week: AUC = 0.84) versus later treatment stages (1–2, 2–4, and 4 weeks: AUC = 0.70, 0.71, and 0.69), reflecting enhanced biological signal capture during initial hepatotoxic response phases prior to adaptive metabolic compensation.

## DISCUSSION

This retrospective analysis of 7,659 voriconazole-treated patients delineates the epidemiological profile and risk factors and outcomes of DILI. The overall DILI incidence was 11.32%, aligning with prior multicenter reports (10.5%–12.5%) (6, 19). Subtype distribution revealed comparable proportions of hepatocellular (5.01%) and cholestatic (5.19%) injuries, with mixed patterns comprising 1.11%, suggesting distinct pathophysiological mechanisms underlying injury phenotypes. Notably, age showed no significant association with subtype distribution, implying predominant roles of genetic or environmental over demographic factors.

Multivariable logistic regression incorporating pharmacokinetic parameters, host pathophysiology, and metabolic biomarkers delineated a multifactorial risk architecture for voriconazole-induced liver injury. First of all, intravenous administration demonstrated the significant association with total liver injury risk (adjusted OR = 1.88, 95% CI: 1.43–2.46), potentially mediated through first-pass metabolism bypass leading to elevated hepatic voriconazole exposure (20). Temporal analysis revealed time-dependent cholestatic injury patterns, with maximal risk during early treatment phases (1–2 weeks: OR = 1.69, 95% CI: 1.08–2.65; 2–4 weeks: OR = 1.94, 95% CI: 1.22–3.09), suggesting dual pathogenic mechanisms involving cumulative toxic metabolite deposition and immune-mediated neoantigen formation during initial drug exposure (21).

Host comorbidity profiling identified baseline hepatic dysfunction (liver cirrhosis, OR = 2.16, 95% CI: 1.58–2.96), diabetes mellitus (OR = 2.19, 95% CI: 1.70–2.80), and transplantation status (hematopoietic stem cell transplantation, OR = 2.93, 95% CI: 1.85–4.65) as critical risk amplifiers. Among these, in transplant populations, there may be pharmacokinetic-immune interactions that result in dual hepatotoxic mechanisms (22). Concurrently, hematopoietic stem cell transplant patients demonstrated monocyte-derived macrophage infiltration during immune reconstitution, establishing a pro-inflammatory microenvironment that amplifies drug-induced oxidative stress in metabolically vulnerable hepatocyte subpopulations (23). Moreover, diabetic pathophysiology appeared to impair hepatocyte autophagy via insulin resistance pathways, reducing compensatory repair capacity (24). The metabolic predictor, which includes elevated baseline urea levels (OR = 1.57, 95% CI: 1.15–2.14), indicated a higher risk of liver injury. This may reflect impaired urea cycle function or a nitrogen metabolic imbalance, both of which can disrupt mitochondrial function and increase susceptibility to liver inflammation and the progression of fibrosis (25).

The association between intravenous administration and hepatocellular injury suggests direct hepatic innate immune activation via pathogen-associated molecular patterns, triggering inflammatory-metabolic cascades (26). Moreover, the observed associations between elevated TG (OR = 1.61, 95% CI: 1.18–2.20) and CRP levels (OR = 1.85, 95% CI: 1.24–2.77) in patients at high risk of cholestatic DILI indicate concurrent dyslipidemia and systemic inflammation may potentiate hepatic lipotoxicity through enhanced lipid synthesis (27).

Based on the study findings, we propose implementing the following clinical strategies for voriconazole administration in high-risk populations, including patients with pre-existing liver disease (especially liver cirrhosis), diabetes mellitus, or organ transplant recipients (particularly hematopoietic stem cell transplantation). Oral administration should be prioritized over intravenous delivery to mitigate hepatic first-pass effects. Although systematic trough monitoring was not performed in our full cohort, subgroup analysis of 138 patients with opportunistically measured levels (Fig. S2) showed no significant difference between control and DILI groups (blue: control, *n* = 100 vs red: DILI, *n* = 38, *P* = 0.7164). This may reflect limitations of single-point measurements in capturing peak toxic exposures or unmeasured metabolites. Nevertheless, therapeutic drug monitoring (TDM) remains recommended per Infectious Diseases Society of America/European Society of Clinical Microbiology and Infectious Diseases (IDSA/ESCMID) guidelines to balance efficacy (AUC/minimal inhibitory concentration [MIC] > 25) and safety given voriconazole’s nonlinear pharmacokinetics. Weekly surveillance of liver function is recommended to enable early liver injury detection. Besides, dose optimization could integrate CYP2C19 genotyping to account for polymorphic metabolic variability (22).

To assess the robustness of our findings, sensitivity analyses were performed after excluding confounding factors. The multivariate-adjusted ORs for primary risk factors remained stable across all models, with little variation in effect estimates. This analytical consistency across multiple covariate adjustments reinforces the validity of our primary conclusions.

While our exclusion criteria ensured methodological rigor for incidence estimation, patients with pre-existing liver injury represent a clinically vulnerable subgroup. In future prospective studies, we would specifically examine voriconazole safety in this population, incorporating pharmacokinetic monitoring (e.g., trough concentrations adjusted for hepatic function), causality assessment tools (e.g., modified Roussel Uclaf Causality Assessment Method [RUCAM] for chronic liver disease), and endpoint definitions for worsening of baseline injury.

Several important limitations warrant consideration when interpreting our findings. First, the exclusive focus on an East Asian population from a single tertiary center may restrict global generalizability. This is particularly relevant given the distinct pharmacoepidemiologic landscape of drug-induced liver injury in Asia-Pacific regions, where both genetic and environmental factors contribute to differential susceptibility profiles. These population-specific characteristics necessitate validation in multi-ethnic cohorts such as the international PRIORITY consortium before broader clinical implementation. Second, while TDM of voriconazole serum trough levels is recommended by current guidelines (IDSA/ESCMID) and could provide additional insights into hepatotoxicity mechanisms, these data found no significant difference between DILI and control groups in our study (Fig. S2). This may reflect methodological constraints of single-point measurements rather than true biological equivalence, as peak-trough fluctuations were unassessed. Third, while CYP2C19 polymorphisms significantly influence voriconazole metabolism and may modify the risk of hepatotoxicity, unfortunately, the exceedingly limited number of patients in our hospital exhibiting genetic polymorphisms of drug-metabolizing enzymes precluded us from obtaining sufficient clinical data for analysis, despite our exhaustive efforts. Despite these limitations, TDM-guided dosing (1 mg/L–5.5 mg/L) remains advised for high-risk subgroups (e.g., cirrhosis, CYP2C19 poor metabolizers) to mitigate hepatotoxicity risk documented in prospective studies. Similarly, CYP2C19 genotyping is prioritized in Asian populations due to higher prevalence of poor metabolizer phenotypes (15%–20% vs 2%–5% in Europeans), which alters voriconazole clearance by four- to fivefold. Future prospective studies incorporating both TDM and pharmacogenetic analyses could help elucidate the relative contributions of pharmacokinetic and pharmacodynamic factors to voriconazole-induced liver injury while validating our identified clinical risk factors.

### Conclusion

In conclusion, our study revealed that the incidence of voriconazole-induced liver injury was significantly influenced by administration route, the host’s metabolic status, and treatment parameters. Among them, intravenous administration was an independent modifiable risk factor, while underlying liver disease (especially liver cirrhosis), diabetes, transplant status (particularly hematopoietic stem cell transplantation), and baseline elevated urea levels as unmodifiable risk factors (elevated CRP and TG levels for cholestatic injury) provide important basis for the identification of high-risk groups and the optimization of treatment regimens.

## Data Availability

All data generated or analyzed during this study is included in this published article.

## SUPPLEMENTAL MATERIAL

Supplemental material aac.00487-25-s0001.docx Tables S1 and S2; Fig. S1 and S2.

ASM does not own the copyrights to Supplemental Material that may be linked to, or accessed through, an article. The authors have granted ASM a non-exclusive, world-wide license to publish the Supplemental Material files. Please contact the corresponding author directly for reuse.
