# Supplementary material for: Voriconazole-induced liver injury: incidence patterns and risk factors in a retrospective cohort
Source: Antimicrob Agents Chemother. 2025 Jul 31;69(9):e00487-25. doi: 10.1128/aac.00487-25 (PMC12406674; doi:10.1128/aac.00487-25)
Supplement: Supplemental material — Tables S1 and S2; Fig. S1 and S2. [file aac.00487-25-s0001.docx]

**Supplementary materials**

**Table S1 Clinical data collection details**

| **Variable** | **Definition** | **Data extraction methods from EHR** |
| --- | --- | --- |
| **Demographics** | | |
| Age | Age when initiating voriconazole therapy. | Extracted from the hospitalization summaries. |
| Gender | Categorized into male and female. |  |
| **Visit information** | | |
| Prior drug allergies | Yes: If patient has allergy history of any prescribed drugs before initiating voriconazole therapy.  No: Otherwise. |  |
| **Administration information of voriconazole** | | |
| Administration route | Categorized into “oral”, “intravenous” and “oral +intravenous” according to the voriconazole prescription records. | Extracted from prescription records within the observation window^*^. |
| VCZ dose per day | Average voriconazole dose (mg) administrated each day within the observation window^*^. |  |
| VCZ Cumulative Time | Cumulative time of the voriconazole administration within the observation window^*^. |  |
| VCZ Cumulative Dose | Cumulative voriconazole dose (mg) administration within the observation window^*^. |  |
| **Pre-medication lab test results** | | |
| ALUBMIN | Alubmin level in plasma | The most recent tests conducted within 30 days before the index date, extracted from the lab test result records in EHR. |
| GLOBULIN | Globulin level in plasma |  |
| HDL | High-density lipoprotein level in serum |  |
| LDL | Low-density lipoprotein level in serum |  |
| TC | Total cholesterol level in serum |  |
| URICACID | Uric acid level in serum |  |
| UREA | Urea level in serum |  |
| CREATININE | Creatinine level in serum |  |
| CRP | C-reactive protein in serum |  |
| **Comorbidities** |  | |
| Liver disease | Yes: If the patient has any diagnosis of hepatitis (including viral hepatitis and other hepatitis), fatty liver disease, liver cirrhosis, liver cancer and other related diseases..  No: If there is explicit statement of no relevant diagnosis.  Unknown: Otherwise. | Extracted from diagnosis records, relevant hospitalization summaries, radiologic imaging reports, and pathology reports within the observation window. For hepatitis B, the information retrieval window was from the first medical visit to the hospital to the end of the observation window. |
| Hepatitis | Yes: If the patient has any diagnosis of varicella hepatitis, chronic hepatitis, viral hepatitis A/B/C, alcoholic hepatitis, and other related diseases.  No: Otherwise | Extracted from diagnosis records, relevant hospitalization summaries, radiologic imaging reports, and pathology reports within the observation window. For hepatitis B, the information retrieval window was from the first medical visit to the hospital to the end of the observation window. |
| Viral hepatitis | Yes: If the patient has any diagnosis of viral hepatitis A/B/C.  No: Otherwise | Extracted from diagnosis records, relevant hospitalization summaries, radiologic imaging reports, and pathology reports within the observation window. For hepatitis B, the information retrieval window was from the first medical visit to the hospital to the end of the observation window. |
| Fatty liver | Yes: If the patient has any diagnosis of fatty liver disease.  No: Otherwise | Extracted from diagnosis records, relevant hospitalization summaries, radiologic imaging reports, and pathology reports within the observation window. |
| Liver cirrhosis | Yes: If the patient has any diagnosis of liver cirrhosis disease.  No: Otherwise | Extracted from diagnosis records, relevant hospitalization summaries, radiologic imaging reports, and pathology reports within the observation window. |
| Liver cancer | Yes: If the patient has any diagnosis of liver cancer disease.  No: Otherwise | Extracted from diagnosis records, relevant hospitalization summaries, radiologic imaging reports, and pathology reports within the observation window. |
| Diabetes mellitus | Yes: If the patient has any diagnosis of diabetes related diseases or has abnormal glucose test results.  No: If there is explicit statement of no relevant diagnosis and no abnormal glucose test results.  Unknown: Otherwise. | Extracted from diagnosis records, relevant hospitalization summaries, and lab test result records from the first medical visit to the hospital to the end of the observation window. |
| Hyperlipidemia | Yes: If the patient has any diagnosis of hyperlipidemia.  No: Otherwise | Extracted from diagnosis records, and relevant hospitalization summaries from the first medical visit to the hospital to the end of the observation window. |
| Hypertension | Yes: If the patient has any diagnosis of hypertension.  No: Otherwise | Extracted from diagnosis records, and relevant hospitalization summaries from the first medical visit to the hospital to the end of the observation window. |
| Hematological diseases | Yes: If the patient has any diagnosis of leukemia, lymphoma, myelodysplastic syndrome. Or if the patient has the lab test results meeting the criteria below:   - serum monoclonal M protein ≥30 g/L, 24 h urine light chain ≥0.5 g - bone marrow monoclonal plasma cell ratio > 10% - tissue biopsy evidence of plasmacytoma   No: If there is explicit statement of no relevant diagnosis.  Unknown: Otherwise | Extracted from diagnosis records, relevant hospitalization summaries, and pathology reports from the first medical visit to the hospital to the end of the observation window. |
| Leukemia | Yes: If the patient has any diagnosis of leukemia.  No: Otherwise | Extracted from diagnosis records, relevant hospitalization summaries, and pathology reports from the first medical visit to the hospital to the end of the observation window. |
| Lymphoma | Yes: If the patient has any diagnosis of lymphoma.  No: Otherwise | Extracted from diagnosis records, relevant hospitalization summaries, and pathology reports from the first medical visit to the hospital to the end of the observation window. |
| Myeloma | Yes: If the patient has any diagnosis of myeloma.  No: Otherwise | Extracted from diagnosis records, relevant hospitalization summaries, and pathology reports from the first medical visit to the hospital to the end of the observation window. |
| Myelodysplastic syndrome | Yes: If the patient has any diagnosis of myelodysplastic syndrome.  No: Otherwise | Extracted from diagnosis records, relevant hospitalization summaries, and pathology reports from the first medical visit to the hospital to the end of the observation window. |
| Transplant state | Yes: If the patient has any hematopoietic stem cell transplantation or organ transplant history.  No: Otherwise | Extracted from diagnosis records, relevant hospitalization summaries, and pathology reports from the first medical visit to the hospital to the end of the observation window. |
| Hematopoietic stem cell transplantation | Yes: If the patient has any hematopoietic stem cell transplant history.  No: Otherwise | Extracted from diagnosis records, relevant hospitalization summaries, and pathology reports from the first medical visit to the hospital to the end of the observation window. |
| Organ transplantation | Yes: If the patient has any organ transplant history, including kidney, liver, lung, bone, corneal, heart, and gut.  No: Otherwise | Extracted from diagnosis records, relevant hospitalization summaries, and pathology reports from the first medical visit to the hospital to the end of the observation window. |
| Kidney transplantation | Yes: If the patient has any kidney transplant history.  No: Otherwise | Extracted from diagnosis records, relevant hospitalization summaries, and pathology reports from the first medical visit to the hospital to the end of the observation window. |
| Liver transplantation | Yes: If the patient has any liver transplant history.  No: Otherwise | Extracted from diagnosis records, relevant hospitalization summaries, and pathology reports from the first medical visit to the hospital to the end of the observation window. |
| Lung transplantation | Yes: If the patient has any lung transplant history.  No: Otherwise | Extracted from diagnosis records, relevant hospitalization summaries, and pathology reports from the first medical visit to the hospital to the end of the observation window. |
| Bone transplantation | Yes: If the patient has any bone transplant history.  No: Otherwise | Extracted from diagnosis records, relevant hospitalization summaries, and pathology reports from the first medical visit to the hospital to the end of the observation window. |
| Corneal transplantation | Yes: If the patient has any corneal transplant history.  No: Otherwise | Extracted from diagnosis records, relevant hospitalization summaries, and pathology reports from the first medical visit to the hospital to the end of the observation window. |
| Heart transplantation | Yes: If the patient has any heart transplant history.  No: Otherwise | Extracted from diagnosis records, relevant hospitalization summaries, and pathology reports from the first medical visit to the hospital to the end of the observation window. |
| Gut transplantation | Yes: If the patient has any gut transplant history.  No: Otherwise | Extracted from diagnosis records, relevant hospitalization summaries, and pathology reports from the first medical visit to the hospital to the end of the observation window. |
| **Combination medications** | | |
| NSAIDs | Yes: If the patient has any prescription of the medications below within 21 days before the index date: sodium salicylate, acetaminophen, ibuprofen, indomethacin, hydroxychloroquine, aspirin.  No: Otherwise. | Extracted from the prescription records within 21 days before the index date. |
| Anti-infective drugs | Yes: If the patient has any prescription of the medications below within 21 days before the index date: rifampicin, pyrazinamide, streptomycin, isoniazid, penicillin, benzylpenicillin, ampicillin, piperacillin, cefazolin, cefuroxime, cefadroxil, cefuroxime, ceftriaxone, cefotiam, amikacin, gentamicin, doxycycline, minocycline, erythromycin, azithromycin, clarithromycin, clindamycin, fosfomycin, compound fosfomycin, amikacin, norfloxacin, ciprofloxacin, levofloxacin, moxifloxacin, metronidazole, tinidazole, sulfapyridine, fluconazole, amphotericin B, itraconazole, acyclovir, ganciclovir, oseltamivir, entecavir, ribavirin, chloroquine, hydroxychloroquine, pyrimethamine, and pyrimethamine.  No: Otherwise. |  |
| Anti-tumor drugs | Yes: If the patient has any prescription of the medications below within 21 days before the index date: iodinated amide, cyclosporine, isocyanuric acid, bleomycin, methotrexate, pyrimidine, cytarabine, fluorouracil, gemcitabine, cisplatin, oxaliplatin, carboplatin, retinoic acid, capecitabine.  No: Otherwise. |  |
| Drugs for central nervous system | Yes: If the patient has any prescription of the medications below within 21 days before the index date: oxcarbazepine, carbamazepine, amantadine, benztropine, aripiprazole, phenytoin, phenobarbital, lamotrigine, haloperidol, clozapine, risperidone, quetiapine, fluoxetine, doxepin, mirtazapine, venlafaxine, diazepam, eszopiclone, zolpidem, midazolam.  No: Otherwise. |  |
| Cardiovascular drugs | Yes: If the patient has any prescription of the medications below within 21 days before the index date: amiodarone, sodium nitroprusside, valsartan, candesartan, ramipril, enalapril, mexiletine, atenolol, nifedipine, diltiazem, propranolol, metoprolol, esmolol, labetalol, felodipine, bosentan, atorvastatin, rosuvastatin, fenofibrate.  No: Otherwise. |  |
| Drugs for Metabolic Diseases | Yes: If the patient has any prescription of the medications below within 21 days before the index date: insulin, metformin, acarbose, liraglutide, repaglinide, pioglitazone, sitagliptin, linagliptin, metformin, propylthiouracil.  No: Otherwise. |  |
| Hormonal drugs | Yes: If the patient has any prescription of the medications below within 21 days before the index date: methyltestosterone, insulin, glargine insulin, tamoxifen, letrozole, thyroid tablets, levothyroxine sodium, ethinyl estradiol, norgestrel.  No: Otherwise. |  |
| Biopharmaceutical | Yes: If the patient has any prescription of the medications below within 21 days before the index date: infliximab, trastuzumab, pemetrexed, interferon B-1a/1b.  No: Otherwise. |  |
| TCM-NM-HP-DS | Yes: If the patient has any prescription of the medications below within 21 days before the index date: Polygonum multiflorum, Peppermint, Radix bupleuri, Astragalus, Common Threewingnut Root, Senna alexandrina Mill., Gynura japonica, Derris trifoliata, Ricinus communis, Xiao Rong Hu Tang, Xiaoyin Pian, Matricaria chamomilla, Humulus scandens.  No: Otherwise. |  |
| * When extracting voriconazole administration information, the observation windows for patients in the DILI group were defined differently from those in the Non-DILI group (as defined in the manuscript). For patients in the DILI group, the observation window started from the index date and extended to the date when hepatotoxicity occurred, and this information was extracted. | | |

**Table S2 Voriconazole treatment duration and DILI incidence**

| **Variable** | **N** | **Frequency** | **DILI incidence** | **value** |
| --- | --- | --- | --- | --- |
| ^a^ Voriconazole Cumulative Time | . | . | . |  |
| ≤1 week | 1407 | 220 | 15.64 | 220(15.64) |
| 1-2 weeks | 1269 | 172 | 13.55 | 172(13.55) |
| 2-3 weeks | 923 | 94 | 10.18 | 94(10.18) |
| 3-4 weeks | 538 | 52 | 9.67 | 52(9.67) |
| 4-5 weeks | 363 | 33 | 9.09 | 33(9.09) |
| 5-6 weeks | 230 | 19 | 8.26 | 19(8.26) |
| 6-8 weeks | 262 | 18 | 6.87 | 18(6.87) |
| 8-12 weeks | 291 | 11 | 3.78 | 11(3.78) |
| 12-16 weeks | 121 | 9 | 7.44 | 9(7.44) |
| >16 weeks | 160 | 2 | 1.25 | 2(1.25) |
| **Variable** | **N** | **Frequency** | **DILI incidence** | **value** |
| ^a^ Voriconazole Cumulative Time | . | . | . |  |
| ≤1 week | 1407 | 220 | 15.64 | 220(15.64) |
| 1-2 weeks | 2676 | 392 | 14.65 | 392(14.65) |
| 2-3 weeks | 3599 | 486 | 13.50 | 486(13.50) |
| 3-4 weeks | 4137 | 538 | 13.00 | 538(13.00) |
| 4-5 weeks | 4500 | 571 | 12.69 | 571(12.69) |
| 5-6 weeks | 4730 | 590 | 12.47 | 590(12.47) |
| 6-8 weeks | 4992 | 608 | 12.18 | 608(12.18) |
| 8-12 weeks | 5283 | 619 | 11.72 | 619(11.72) |
| 12-16 weeks | 5404 | 628 | 11.62 | 628(11.62) |
| >16 weeks | 5564 | 630 | 11.32 | 630(11.32) |

^a^ voriconazole cumulative days: Cumulative days of voriconazole treatment from the time of first administration to the date of DILI occurred

**Figure S1 Crude Univariable Odds Ratios (ORs) for Risk Factors Associated With DILI and Its subtypes.**

**Abbreviations:** VCZ: voriconazole; HDL: high-density lipoprotein; LDL: low-density lipoprotein; TC: total cholesterol; TG: triglycerides; URICACID: uric acid; CRP: C-reactive protein; NSAIDs: Nonsteroidal Antiinflammatory Drugs; TCM-NM-HP-DS: Traditional Chinese Medicine-Natural Medicine-Health Products-Dietary Supplements

**Reference levels**: Age(years): <40; Gender: Male; Administration route: oral; VCZ Dose Per Day: ≤200 mg; VCZ Cumulative time: ≤1 week; Prior drug allergies, Liver disease, Viral hepatitis, Fatty liver, Liver cirrhosis, Liver cancer, Hepatitis, Diabetes mellitus, Hyperlipidemia, Hypertension, Hematological diseases, Leukemia, Lymphoma, Myeloma, Myelodysplastic syndrome, Transplant state, Hematopoietic Stem Cell Transplantation, Organ Transplantation, Nonsteroidal Antiinflammatory Drugs, NSAIDs, Anti-infective drugs, Anti-tumor drugs, Drugs for central nervous system, Cardiovascular drugs, Drugs for Metabolic Diseases, Hormonal drugs, Biopharmaceutical and TCM-NM-HP-DS: No; ALBUMIN, GLOBULIN, HDL, LDL, TC, TG, URICACID, UREA, CREATININE and CRP: normal.


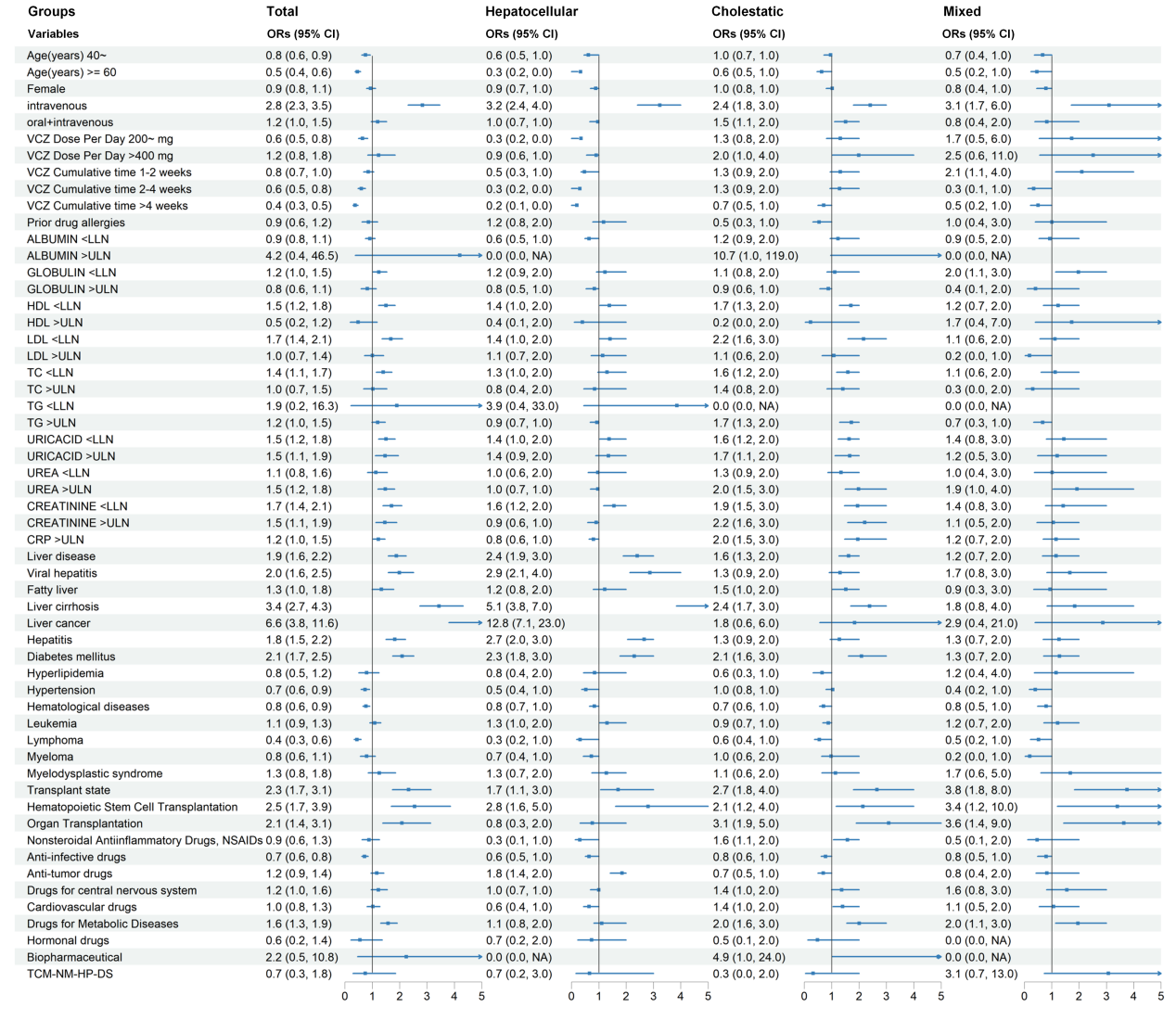


**Figure S2 Comparison of voriconazole blood concentration levels between the control group and the liver injury group (n = 100 in the control group, n = 38 in the DILI group).**

**
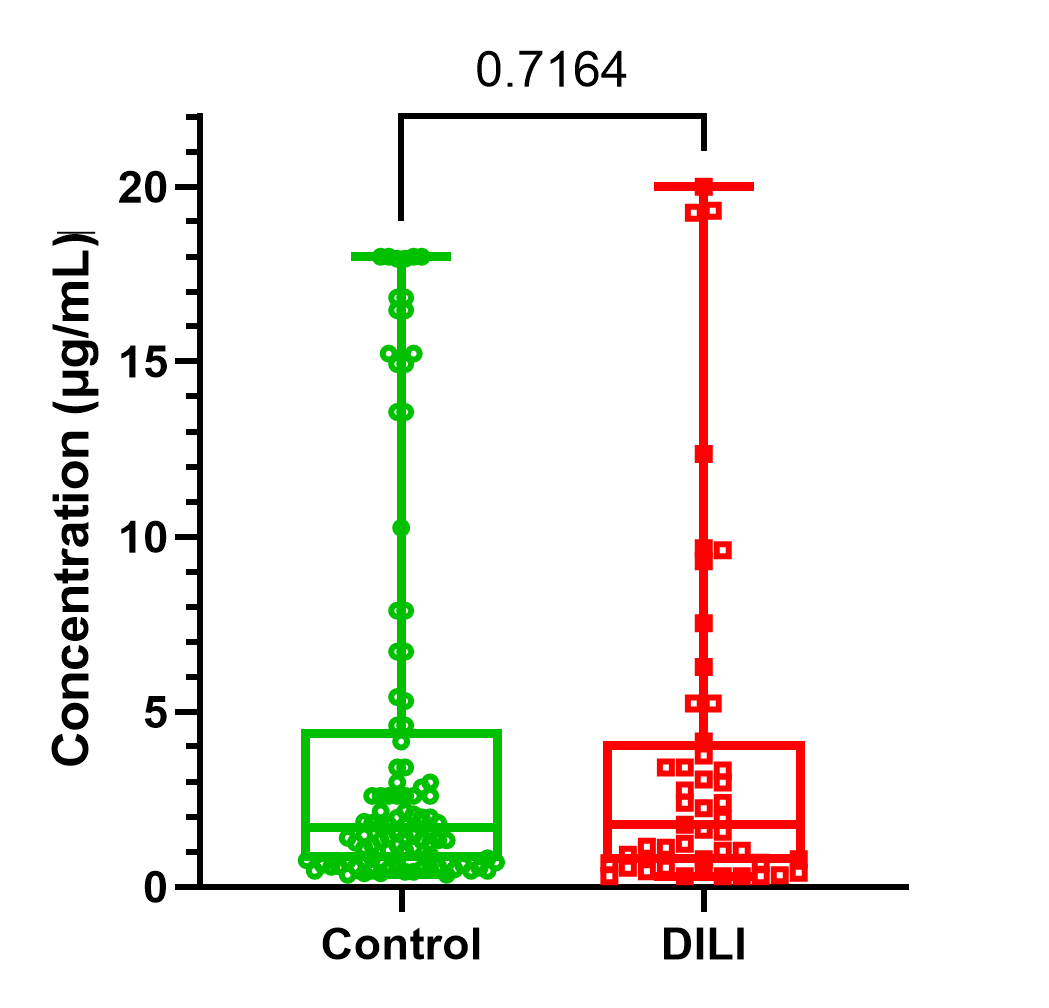
**
